# Evidence of Strange Attractors in Class C Amplifier with Single Bipolar Transistor: Polynomial and Piecewise-Linear Case

**DOI:** 10.3390/e23020175

**Published:** 2021-01-30

**Authors:** Jiri Petrzela

**Affiliations:** Department of Radio Electronics, Faculty of Electrical Engineering and Communication, Brno University of Technology, Technicka 12, 616 00 Brno, Czech Republic; petrzelj@feec.vutbr.cz; Tel.: +420-54114-6561

**Keywords:** admittance parameters, bipolar transistor, entropy, chaos, Lypunov exponent, non-unilateral two-port, strange attractors

## Abstract

This paper presents and briefly discusses recent observations of dynamics associated with isolated generalized bipolar transistor cells. A mathematical model of this simple system is considered on the highest level of abstraction such that it comprises many different network topologies. The key property of the analyzed structure is its bias point since the transistor is modeled via two-port admittance parameters. A necessary but not sufficient condition for the evolution of autonomous complex behavior is the nonlinear bilateral nature of the transistor with arbitrary reason that causes this effect. It is proved both by numerical analysis and experimental measurement that chaotic motion is miscellaneous, robust, and it is neither numerical artifact nor long transient motion.

## 1. Introduction

Chaos can be considered as long-time unpredictable behavior of a dynamical system that is both nonlinear and, in the autonomous case, has at least three degrees of freedom. Chaotic systems are very sensitive to the changes of initial conditions; this sensitivity is caused by exponential divergency of neighborhood orbits but, at the same time, the generated strange attractor is bounded into finite state space volume. The boundedness of the strange attractor is due to the suitable nonlinearity of the vector field. Despite mature observations, recent studies reveal that fixed points are not crucial for the evolution of chaos. There are several mathematical models with equilibrium degenerated into higher-dimensional geometric structures or chaotic systems without equilibrium. Additionally, a wide variety of chaotic dynamical systems that exhibit the so-called hidden attractors are available via internet search.

After its analytical, numerical, and experimental confirmation within a very simple fully analog circuit, famous Chua’s oscillator [1], chaos started to receive considerable attention, especially among circuit design engineers. Many interesting theories and practical findings coupled with nonlinear dynamics in lumped circuits were discovered and published; several examples can be found in papers [2,3,4,5,6,7,8]. By following subsequent discoveries in chaos theory and by increasing the knowledge of the strange attractor´s evolution, this kind of complex motion was detected in many electronic systems. Let us mention a few cases of naturally non-chaotic building blocks of more complex systems dedicated for analog signal processing. Robust chaos was reported in switched capacitor circuits [9], switched regulators [10], power converters [11], different topologies of dc-dc converters [12], and in power electronics in general. From the viewpoint of subsystems of radio-frequency path, structurally stable chaotic oscillations were discovered in phase-locked loops [13], multi-state memory cells [14,15], and standard structures of harmonic oscillators such as Colpitts [16], Hartley [17], Wien-bridge [18] or other topology having resistor-capacitor feedback [19] topology.

Recent papers [20,21] have revealed the existence of strange attractors in a fundamental stage of class C amplifier with a single bipolar transistor. However, the bipolar transistor in this paper is assumed to have linear forward trans-conductance *y*_21_(*v*_1_), while work [20] assumes a cubic polynomial for both functions *y*_12_(*v*_2_), *y*_21_(*v*_1_). Therefore, all seven distinct chaotic cases revealed in this manuscript are algebraically simpler than the single example proposed in paper [20]. In contrast, paper [21] deals with smooth nonlinear function *y*_21_(*v*_1_) and presents strange attractors discovered for only two shapes of forward trans-conductance. On the other hand, in the upcoming analysis, smooth polynomial nonlinearity up to the fifth order that describes backward trans-conductance could be found within the set of the ordinary differential equations. Of course, the different mathematical model considered here leads to completely different numerical results as well as much simpler circuitry implementation. From the practical perspective, the third-order deterministic chaotic systems provided in this paper generate waveforms with different features. Readers can pick and use our dynamical system that fits a specific application. In general, the upcoming sections represent more comprehensive analysis of the class C amplifier than case study [20]. Simple circuits with one or two transistors are analyzed in paper [22], again from the viewpoint of the evolution of chaotic behavior. In these networks, the parasitic properties of transistors are not considered for the numerical investigations.

Driven lumped electronic systems are subjects of chaotic dynamics as well. Moreover, degrees of freedom can be lowered to two. The chaotic operational regime of a KHN (Kerwin–Huelsman–Newcomb) filter (or state variable filters in general) is analyzed in paper [23]. It is demonstrated that chaos occurs and disappears according to the frequency and amplitude of input useful harmonic signal. Based on the observations presented in this paper, a two-terminal electronic device marked as “chaotic admittance” can be developed. While applied input voltage acts as a driving force, the chaotic waveform measured at any independent internal node controls input current. The practical application of such two-terminal can be discovered in noise generators, the testing of frequency responses, analog and digital modulations, the masking of useful analog signals using chaotic waveform [24], etc.

## 2. Single Transistor Stage

**Figure 1:**
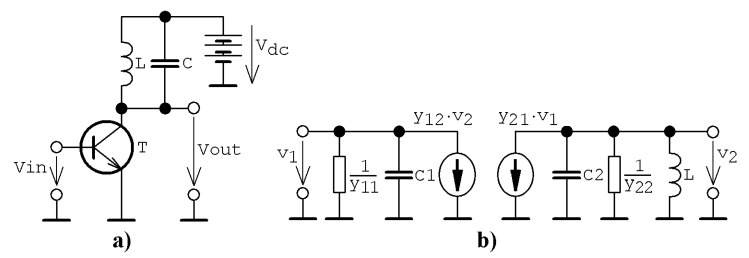
General circuit concepts analyzed in this paper: (**a**) fundamental cell of class C amplifier, (**b**) equivalent schematic of class C amplifier for useful small-amplitude AC (Alternating Current) signals.

Assume the lumped electronic equivalent of a single transistor that is characterized by arbitrary bias point and connected as indicated in Figure 1a. For a useful signal, a simplified small-signal calculation model provided in Figure 1b can be derived. Describing a set of ordinary differential equations can be expressed in matrix form as:

(1) $$\frac{d}{dt}x=A\cdot x\;↔\;\frac{d}{dt}\left(\begin{array}{c} v_{1}\\ v_{2}\\ i_{\ell } \end{array}\right)=\left(\begin{array}{ccc} -\frac{y_{11}}{c_{1}} & -\frac{y_{12}}{c_{1}} & 0\\ -\frac{y_{21}}{c_{2}} & -\frac{y_{22}}{c_{2}} & -\frac{1}{c_{2}}\\ 0 & \frac{1}{\ell } & 0 \end{array}\right)\cdot \left(\begin{array}{c} v_{1}\\ v_{2}\\ i_{\ell } \end{array}\right),$$

where *y_jk_* are admittance parameters of a bipolar transistor considered as two-port in a common emitter configuration and the state vector is $x={\left(v_{1},\;v_{2},\;i_{\ell }\right)}^{T}$. Symbol *c*_1_ represents a parasitic base-emitter capacitance and *c*_2_ is a sum of parasitic collector-emitter capacitance and working capacitance of the parallel resonant tank. Resistor *R*_2_ given in the schematic could contain both the output admittance of transistor *y*_22_ and inductance resistive losses. In practice, entries of 2 × 2 transistor´s admittance matrix could be complex numbers, especially if high-frequency applications are addressed. It is necessary to realize that the word “high” is relative—it could be tens of MHz depending on the type of bipolar transistor. Coefficients of the transistor´s admittance matrix could also be nonlinear functions, specifically in the case of power amplifiers or if signals with high amplitudes are processed.

Of course, numerical values of admittance parameters depend significantly on biasing circuitry. These are not specified in the analyzed schematic. However, to maintain the maximum universality of final remarks, the input and output admittance of a bipolar transistor is supposed to be linear, i.e., constant real number independent of the amplitude of a processed signal. The fundamental amplification capability of a bipolar transistor characterized by trans-admittance *y*_21_ will be scalar constant as well, which is a linear function of input voltage *v*_1_ without offset. Therefore, a characteristic polynomial associated with the isolated system (1) and evaluated at the fixed point $x_{e}={\left(v_{1}^{0},\;v_{2}^{0},\;i_{\ell }^{0}\right)}^{T}$ is:

(2) $$\det\left(\gamma \cdot E-A\right)=\gamma^{3}+\frac{c_{2}\cdot y_{11}+c_{1}\cdot y_{22}}{c_{1}\cdot c_{2}}\cdot \gamma^{2}+\frac{1}{c_{1}\cdot c_{2}\cdot \ell }\left(c_{1}+\ell \cdot y_{11}\cdot y_{22}-\ell \cdot y_{21}\cdot {\frac{\partial y_{12}}{\partial v_{2}}|}_{v_{2}^{0}}\right)\cdot \gamma +\frac{y_{11}}{c_{1}\cdot c_{2}\cdot \ell }=0\;,\;$$

where **E** is the unity matrix. A partial derivative of backward trans-conductance of a bipolar transistor is evaluated at equilibrium structure *d***x**/*dt* = **0**, where **0** is a vector of zeroes. Note that, at this moment, accumulation elements are normalized with respect to time and impedance. This fact is emphasized by utilization of the small letters $c_{1},\;c_{2},\ell$ throughout this manuscript.

### 2.1. Local Polynomial Backward Trans-Conductance

Let us rewrite the matrix system of differential Equation (1) into the more general form that considers the possible fractional-order nature of the individual accumulation elements and a polynomial backward trans-conductance of a bipolar transistor. New ordinary differential equations will be:

(3) $$\frac{d^{\alpha }}{dt^{\alpha }}v_{1}=-\frac{y_{11}}{c_{1}}v_{1}-\frac{1}{c_{1}}\left(a\cdot v_{2}+b\cdot v_{2}^{2}+c\cdot v_{2}^{3}+d\cdot v_{2}^{4}+e\cdot v_{2}^{5}\right),\;\frac{d^{\beta }}{dt^{\beta }}v_{2}=-\frac{y_{21}}{c_{2}}v_{1}-\frac{y_{22}}{c_{2}}v_{2}-\frac{1}{c_{2}}i_{\ell },\;\frac{d^{\gamma }}{dt^{\gamma }}i_{\ell }\;=\;\frac{1}{\ell }v_{1}\;,$$

where orders *α*, *β*, and *γ* are real numbers between zero and one. In this case, system (1) has one fixed point located at the origin. Firstly, let us consider cases where coefficient *a* = 0. Eigenvalues, i.e., solutions of cubic polynomial (2) associated with $x_{e}={\left(0,\;0,\;0\right)}^{T}$, imply that the origin is a non-repelling fixed point, at least for reasonable values of transistor cell components, i.e., nonzero positive *c*_1_, *c*_2_, ℓ and positive system dissipation, i.e., *y*_11_ > 0 and *y*_22_ > 0.

(4) $$\gamma_{1}=-\frac{y_{11}}{c_{1}}\;,\;\gamma_{2,3}=-\frac{1}{2\cdot c_{2}}\cdot \left(y_{22}\mp \sqrt{y_{22}^{2}-4\frac{c_{2}}{\ell }}\right)\;,$$

Therefore, all nontrivial solutions including sought strange attractors belong to the so-called hidden attractors [25]. The local vector field near to the origin is spanned by the eigenvector $\vec{\varepsilon_{1}}$ and the eigenplane defined by $\vec{\varepsilon_{2,3}}$ written in the following symbolic form:

(5) $$\vec{\varepsilon_{1}}=\left(\begin{array}{c} -\frac{c_{1}^{2}-\ell \cdot c_{1}\cdot y_{11}\cdot y_{22}+c_{2}\cdot \ell \cdot y_{11}^{2}}{c_{1}^{2}\cdot y_{21}}\\ -\frac{\ell \cdot y_{11}}{c_{1}}\\ 1 \end{array}\right)\;,\;\vec{\varepsilon_{2,3}}=\left(\begin{array}{c} 0\\ -\frac{\ell \cdot y_{22}\mp \sqrt{y_{22}^{2}-4\frac{c_{2}}{\ell }}}{2\cdot c_{2}}\\ 1 \end{array}\right)\;.$$

As it will be clarified later, case *a* = 0 covers four out of six discovered sets of parameters that lead to robust chaotic behavior. Now assume an arbitrary value of coefficient *a*. By following Cardan´s rule, one can obtain the following symbolic eigenvalues:

(6) $$\gamma =-\frac{1}{3\cdot \beta }\left[\frac{1}{c_{2}\cdot \ell }-\frac{a\cdot y_{21}}{c_{1}\cdot c_{2}}+\frac{y_{11}\cdot y_{22}}{c_{1}\cdot c_{2}}-\frac{1}{3}{\left(\frac{y_{11}}{c_{1}}+\frac{y_{22}}{c_{2}}\right)}^{2}\right]+\beta -\frac{1}{3}\left(\frac{y_{11}}{c_{1}}+\frac{y_{22}}{c_{2}}\right)\;,$$

where the first auxiliary parameter:

(7) $$\beta =\sqrt[{3}]{-\frac{\vartheta }{2}\pm \sqrt{\frac{\vartheta^{2}}{4}+\frac{{\left[\frac{1}{c_{2}\cdot \ell }-\frac{a\cdot y_{21}}{c_{1}\cdot c_{2}}+\frac{y_{11}\cdot y_{22}}{c_{1}\cdot c_{2}}-\frac{1}{3}{\left(\frac{y_{11}}{c_{1}}+\frac{y_{22}}{c_{2}}\right)}^{2}\right]}^{3}}{27}}}\;,$$

and the second auxiliary parameter can be calculated as:

(8) $$\vartheta =\frac{y_{11}}{\ell \cdot c_{1}\cdot c_{2}}+\frac{1}{27}\left[2{\left(\frac{y_{11}}{c_{1}}+\frac{y_{22}}{c_{2}}\right)}^{3}-9\left(\frac{y_{11}}{c_{1}}+\frac{y_{22}}{c_{2}}\right)\left(\frac{1}{c_{2}\cdot \ell }-\frac{a\cdot y_{21}}{c_{1}\cdot c_{2}}+\frac{y_{11}\cdot y_{22}}{c_{1}\cdot c_{2}}\right)\right]\;.$$

For conservative dynamics, i.e., if *y*_11_ = *y*_22_ = 0, formulas for the eigenvalues (6), (7) and (8) significantly simplify into the following relation:

(9) $$\gamma_{1,2}=\pm \sqrt{\frac{\ell \cdot a\cdot y_{21}-c_{1}}{\ell \cdot c_{1}\cdot c_{2}}}\;,\;\gamma_{3}=0.$$

A closer insight into these eigenvalues is not necessary since the optimization routine (see below) operates with numerical values of network elements and, consequently, with numerical eigenvalues.

Although case *a* ≠ 0 was considered during the searching-for-chaos procedure, its linearized analysis is not provided in this paper. Corresponding symbolic formulas are too complicated to be displayed using a reasonable format.

### 2.2. Local Piecewise-Linear Backward Trans-Conductance

The idea behind this sub-section is to assume the successful (in the sense of comparable values of positive largest Lyapunov exponent) approximation of backward trans-conductance *y*_12_(*v*_2_) by two- or three-segment piecewise linear (PWL) curves. A set of three ordinary differential equations that describe the class C amplifier is:

(10) $$\frac{d^{\alpha }}{dt^{\alpha }}v_{1}=-\frac{y_{11}}{c_{1}}v_{1}-\frac{1}{c_{1}}y_{12}\left(v_{2}\right),\;\frac{d^{\beta }}{dt^{\beta }}v_{2}=-\frac{y_{21}}{c_{2}}v_{1}-\frac{y_{22}}{c_{2}}v_{2}-\frac{1}{c_{2}}i_{\ell }\;,\;\frac{d^{\gamma }}{dt^{\gamma }}i_{\ell }=\frac{1}{\ell }v_{1}$$

where *α*, *β*, and *γ* are real numbers between zero and one. The approximated vector field is symmetrical with respect to origin. Therefore, for odd-symmetrical PWL function, backward trans-conductance with four breakpoints {−*ϕ*_2_, −*ϕ*_1_, *ϕ*_1_, *ϕ*_2_} can be expressed as:

(11) $$y_{12}\left(v_{2}\right)=\rho_{2}\cdot x+\frac{\rho_{0}-\rho_{1}}{2}\cdot \left(\left|x+\varphi_{1}\right|-\left|x-\varphi_{1}\right|\right)+\frac{\rho_{1}-\rho_{2}}{2}\cdot \left(\left|x+\varphi_{2}\right|-\left|x-\varphi_{2}\right|\right)\;,$$

where *ρ*_0_ is slope of segment around zero, *ρ*_1_ is slope of segment between breakpoint *ϕ*_1_ and *ϕ*_2_ (*ϕ*_1_ < *ϕ*_2_), and *ρ*_2_ is slope of segment in the outer regions of the vector field, i.e., *x* > *ϕ*_2_. For even-symmetry of the vector field, PWL function could possess three breakpoints {−*ϕ*, 0, *ϕ*} and be characterized by the following simple relation:

(12) $$y_{12}\left(v_{2}\right)=\rho_{0}\cdot \left|x\right|+\frac{\rho_{1}-\rho_{0}}{2}\cdot \left(\left|x+\varphi \right|+\left|x-\varphi \right|\right)-\varphi \cdot \left(\rho_{1}-\rho_{0}\right).$$

Obviously, such PWL function has slope *ρ*_0_ for 0 < *x* < *ϕ*, slope −*ρ*_0_ for −*ϕ* < *x*< *0*, slope −*ρ*_1_ for *x* < −*ϕ*, and finally slope *ρ*_1_ for *x* > *ϕ*. Both PWL functions generate a single equilibrium point located at the origin and the entire vector field is separated into five and four affine segments for odd (11) and (12) even-symmetrical PWL function, respectively. Note that the existence of other fixed points is not conditioned by the shape of PWL functions. In other words, for scalar PWL functions, there is one equilibrium point located at ${\left(-y_{12}\left(0\right)/y_{11},\;0,\;y_{21}\cdot y_{12}\left(0\right)/y_{11}\right)}^{T}$. Assume constant term *ρ_const_* in PWL function (11) or (12). Then, *ρ_const_* can be used to move the equilibrium point to a new position in the state space along a line.

### 2.3. Alternative Mathematical Models of Class C Amplifier

So far, a bipolar transistor substituted by two-port described by admittance matrix was considered to be a dynamical system dedicated for analysis. Firstly, note that a transistor cannot be directly substituted by the very popular Giacoletto´s model or a similar interconnection where backward trans-conductance is neglected. Secondly, an arbitrarily biased bipolar transistor can be modeled using other types of two-port equivalent parameters, such as impedance or hybrid matrix. However, this change does not bring benefits over the initial admittance matrix **Y**; neither from the viewpoint of linear analysis nor circuitry realization. For a bipolar transistor modeled by impedance matrix **Z** = **Y**^−1^ we can obtain the following algebraic relations:

(13) $$z_{11}=\frac{y_{22}}{y_{11}\cdot y_{22}-y_{12}\cdot y_{21}}=0\;,\;z_{12}=-\frac{y_{12}}{y_{11}\cdot y_{22}-y_{12}\cdot y_{21}}=1\;,\;z_{21}=-\frac{y_{21}}{y_{11}\cdot y_{22}-y_{12}\cdot y_{21}}=\frac{1}{y_{12}},\;z_{22}=\frac{y_{11}}{y_{11}\cdot y_{22}-y_{12}\cdot y_{21}}=-\frac{y_{11}}{y_{12}}\;,$$

where significant simplifications provided above are valid for a zero output admittance *y*_22_ = 0 and a normalized forward trans-conductance *y*_21_ = 0. Analogically, for a bipolar transistor described by the hybrid matrix, we can obtain:

(14) $$h_{11}=\frac{1}{y_{11}}\;,\;h_{12}=-\frac{y_{12}}{y_{11}}\;,\;h_{21}=\frac{1}{y_{11}}\;,\;h_{22}=\frac{y_{11}\cdot y_{22}-y_{12}\cdot y_{21}}{y_{11}}=-\frac{y_{12}}{y_{11}}\;,\;$$

where the combination of *y*_22_ = 0 and *y*_21_ = 1 provides simplification again. Obviously, a chaotic system based on a class C amplifier can be constructed using (13) and (14), or by introducing a linear transformation of coordinates applied on system (3) or (10).

A single-transistor class C amplifier can be also modeled by a two-port equivalent circuit of a bipolar transistor where three two-terminal devices (initially admittances) are arranged into Π topology appended by one voltage controlled current source. This controlled source can be located at the input port, output port or between these ports. However, no such transformation reduces the complexity of the final circuit since much more complex polynomial nonlinearities need to be implemented as lumped electronic subcircuits. From the viewpoint of global dynamics, the Π-type configuration of an equivalent circuit can still generate robust chaotic waveforms.

### 2.4. Searching for Chaotic Case

For the process of chaos localization using the largest Lyapunov exponent as the objective function [26], transfer characteristics of backward trans-conductance were approximated by a polynomial up to the sixth order. Finally, it turns out that the values of both capacitors and inductors can be kept constant (unity) during optimization without losing the chance to find a chaotic solution. Additionally, a bipolar transistor is supposed to work close to ideal current source with *y*_22_→0 and forward trans-conductance *y*_21_ = 1 S. Normalized eigenvalues associated with the origin will be γ_1_ = −*y*_11_, γ_2,3_ = ±j, i.e., neighborhood trajectories are attracted to an eigenplane where limit cycle is evolved. This is a quite unusual situation in chaos theory. Therefore, the sixth-dimensional hyperspace of the internal parameters of a dynamical system (1) with the edges **Ψ**∈{*y*_11_, *a*, *b*, *c*, *d*, *e*} undergoes deep investigation. The last five parameters shape nonlinear feedback function (3).

Since individual points in this hyperspace can be calculated independently (an arbitrary number of the fitness functions can be calculated simultaneously), Matlab and CUDA-based parallel processes represent a good choice for high precision and fast calculation. Objective function is a combination of three phenomena: a bounded state attractor (verified during numerical integration), a positive value of LLE (taken as a final value after integration), and predefined local geometry near the fixed point. Since there is no closed-form analytic solution associated with chaotic dynamics, stochastic nature-inspired optimization (a combination of genetic algorithm and swarm intelligence) was utilized. So far, several configurations **Ψ** with a reasonable (from the viewpoint of potential practical applications involving experimental construction of the chaotic circuit) seven-sided volume have been found. More details can be found in Table 1 for polynomial vector field and Table 2 for PWL case. The provided cases represent differently shaped (in the geometric sense) strange attractors and this list is by no means complete.

Dynamical system (1) can be rewritten in the form of a jerk function, that is, as the third order differential equation, namely:

(15) $$\frac{d^{3}}{dt^{3}}i_{L}+\left(\frac{y_{11}}{c_{1}}+\frac{y_{22}}{c_{2}}\right)\cdot \frac{d^{2}}{dt^{2}}i_{L}+\left(\frac{1}{c_{2}\cdot L}+\frac{y_{11}\cdot y_{22}}{c_{1}\cdot c_{2}}-\frac{y_{12}\cdot y_{21}}{c_{1}\cdot c_{2}}\right)\cdot \frac{d}{dt}i_{L}+\frac{y_{11}}{c_{1}\cdot c_{2}\cdot L}\cdot i_{L}=0\;,\;$$

or a simplified form by considering all assumptions provided above:

(16) $$\frac{d^{3}}{dt^{3}}i_{L}+y_{11}\cdot \frac{d^{2}}{dt^{2}}i_{L}+\left(1-y_{12}\cdot y_{21}\right)\cdot \frac{d}{dt}i_{L}+y_{11}\cdot i_{L}=0\;.$$

In both (15) and (16), *y*_12_ is a nonlinear scalar function of variable *v*_2_. A single higher-order differential equation has a simple circuit representation: cascade connection of integrators with two-port feedback branches and an input summation/differentiation stage.

Third-order differential Equation (15) or (16) can be instantaneously compared with the so-called jerk functions discovered during the excessive search performed by several scientists in the most recent three decades. Prof. Sprott was especially active in this research field and discovered many algebraically simple dynamical systems with a chaotic solution [27,28,29]. From this perspective, mathematical model (1) with parameters **Ψ**_1_ up to **Ψ**_7_ represents a new chaotic system that cannot be transformed into some known third-order system via a linear change of coordinates.

## 3. Numerical Results

**Figure 2:**
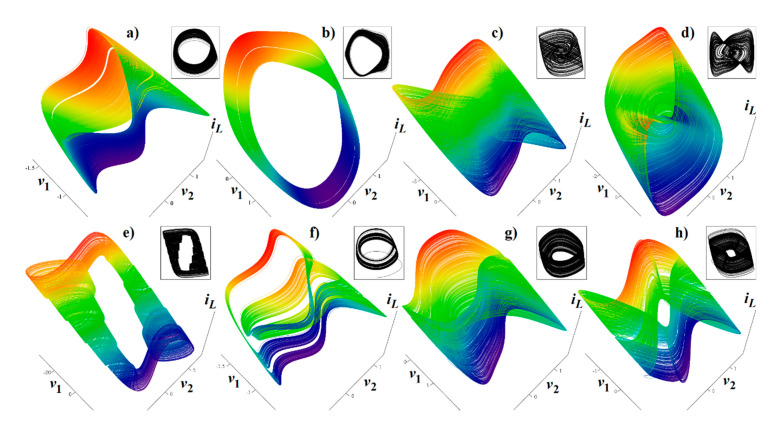
Plane projection *v*_1_ vs. *v*_2_ (black) and rainbow colored three-dimensional perspective views on the typical strange attractors generated by: (**a**) parameter set **Ψ**_1_ substituted into the expression (1), (**b**) parameter set **Ψ**_1_ substituted into system (6), (**c**) parameter set **Ψ**_2_ substituted into differential Equation (1), (**d**) parameter set **Ψ**_2_ substituted into jerk dynamics (6), (**e**) parameter set **Ψ**_3_ numerically integrated using Equation (1), (**f**) integration of system (1) with parameter set **Ψ**_4_, (**g**) parameter set **Ψ**_5_ substituted into Equation (1), and (**h**) parameter set **Ψ**_6_ substituted into system (1) and integrated.

The core engine for all routines used for numerical analysis presented in this paper is a fourth order Runge–Kutta method with fixed step size. The numerical integration of both mathematical descriptions of the discovered chaotic system, i.e., matrix expression (1) and its normal form, calculated in Mathcad is provided by means of Figure 2. For this fundamental analysis, final time was set to 10,000 s, time step was 0.01 s, initial conditions were chosen **x**_0_ = (−1, 0, 0)^T^ for parameter set **Ψ**_1,2,3,5,6_ and **x**_0_ = (2, 0, 0)^T^ for parameter set **Ψ**_4_. Individual cases of parameters **Ψ**_1–6_ are associated with Table 1.

**Figure 3:**
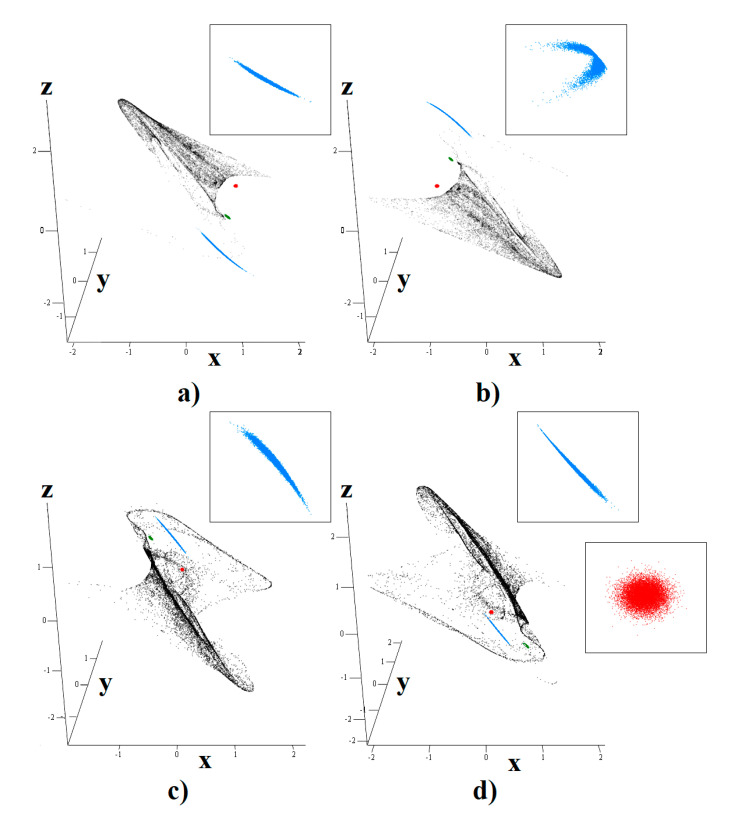
Sensitivity to tiny changes of initial condition demonstrated for first case of chaotic system: starting situation (red points), short time evolution (green points), average time evolution (blue dots) and long time separation (black dots). Nominal starting position is chosen as follows: (**a**) **x**_0_ = (1, 0, 0)^T^, (**b**) **x**_0_ = (−1, 0, 0)^T^, (**c**) **x**_0_ = (0, −1, 0)^T^ and (**d**) **x**_0_ = (0, 1, 0)^T^. Magnified areas showing states are demonstrated.

Figure 3 shows that the chaotic system is extremely sensitive to the small variations of initial states, as required for the chaotic dynamics. This kind of analysis was performed for the **Ψ**_1_ case of the chaotified class C amplifier (see Table 1), but similar results can be obtained for the rest of the system cases, both polynomial and PWL. In these graphs, red dots represent 10^4^ initial conditions with normal distribution, standard deviation 0.01 and nominal value **x**_0_ = (1, 0, 0)^T^. Other colors have the following meanings: final state is stored after 1 s (green points), ending state after 10 s (blue dots) and final state after 100 s (black dots). Note that neighborhood trajectories diverge slowly, and after 10 s fiducial points are still closely spaced. There is one exception: system case **Ψ**_2_ possesses the higher degree of long-time unpredictability.

**Figure 4:**
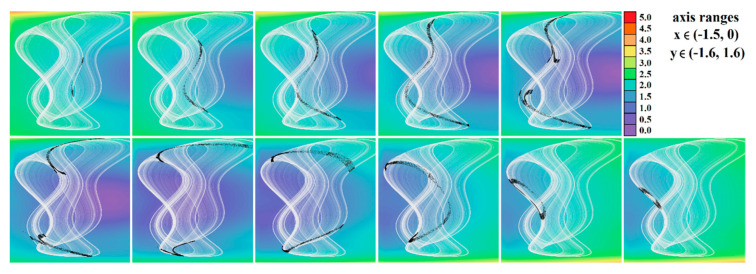
Horizontal state space slices given by *z* = *const*. showing kinetic energy distribution of typical chaotic attractors of case **Ψ**_1_ system, associated Poincaré sections (black dots). Figures sorted from left to right and up to down: *z* = −0.9, *z* = −0.7, *z* = −0.5, *z* = −0.2, *z* = 0, *z* = 0.3, *z* = 1, *z* = 1.5, *z* = 2, *z* = 2.4, and *z* = 2.47.

**Figure 5:**
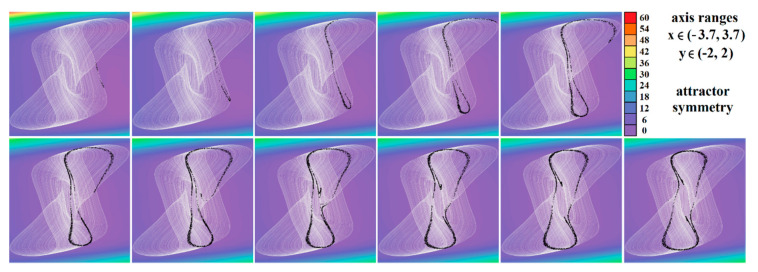
Horizontal state space slices defined by the plane *z = const*. and providing dynamical energy distribution of typical chaotic attractors of case **Ψ**_2_ system (white curve), associated Poincaré sections (black dots). Figures sorted from left to right and up to down: *z =* −3.3, *z =* −3, *z =* −2.5, *z =* −2, *z =* −1.5, *z =* −1, *z =* −0.8, *z =* −0.6, *z =* −0.4, *z =* −0.2, and *z =* 0.

**Figure 6:**
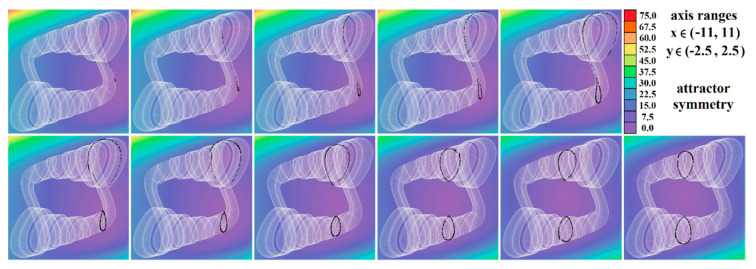
Horizontal state space slices given by plane *z = const*. providing rainbow scaled dynamical energy distribution of the typical chaotic attractors of case **Ψ**_3_ system, associated Poincaré sections (black dots). Individual figures are sorted from left to right and up to down with respect to the planes: *z =* −9.4, *z =* −9, *z =* −8.5, *z =* −8, *z =* −7, *z =* −6.5, *z =* −6, *z =* −4, *z =* −2, *z =* −1, and *z =* 0.

**Figure 7:**
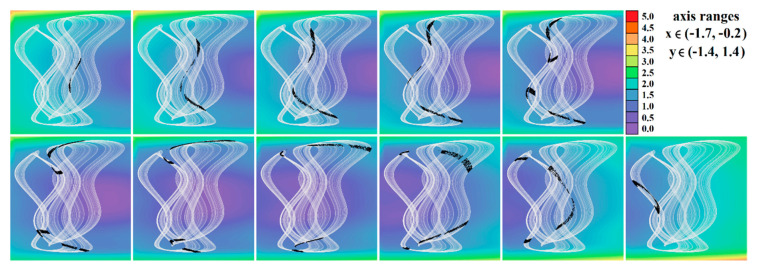
Horizontal state space slices given by plane *z = const*. providing rainbow scaled dynamical energy distribution of the typical chaotic attractors of case **Ψ**_4_ system (white trajectory), and associated Poincaré sections (black dots). Figures sorted from left to right and up to down: *z =* −0.4, *z =* −0.2, *z =* 0, *z =* 0.2, *z =* 0.3, *z =* 0.5, *z =* 0.8, *z =* 1.2, *z =* 1.6, *z =* 2, and *z =* 2.5.

**Figure 8:**
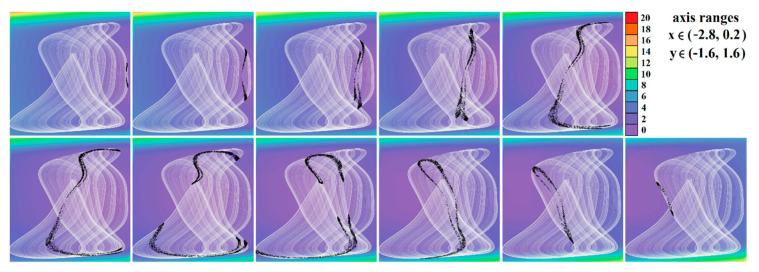
Horizontal state space slices given by plane *z = const*. providing rainbow scaled dynamical energy distribution of the typical chaotic attractors of case **Ψ**_5_ system (white trajectory), and associated Poincaré sections (black dots). Individual figures are sorted from left to right and up to down: *z =* −1, *z =* −0.8, *z =* −0.6, *z =* −0.2, *z =* 0.4, *z =* 0.6, *z =* 1, *z =* 1.5, *z =* 2, *z =* 2.5, and *z =* 2.9.

**Figure 9:**
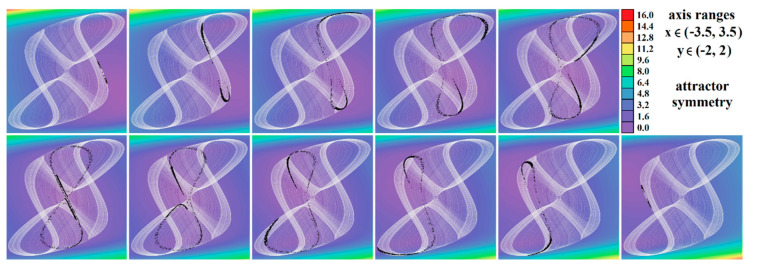
Horizontal state space slices defined by plane *z = const*. providing rainbow scaled dynamical energy distribution of typical chaotic attractors of case **Ψ**_6_ system (white state trajectory), associated Poincaré sections (black dots). Figures sorted from left to right and up to down are given by: *z =*−3.3, *z =* −2.7, *z =* −2, *z =* −1.2, *z =* −0.7, *z =* 0, *z =* 0.4, *z =* 1, *z =* 1.7, *z =* 2.3, and *z =* 3.3.

**Figure 10:**
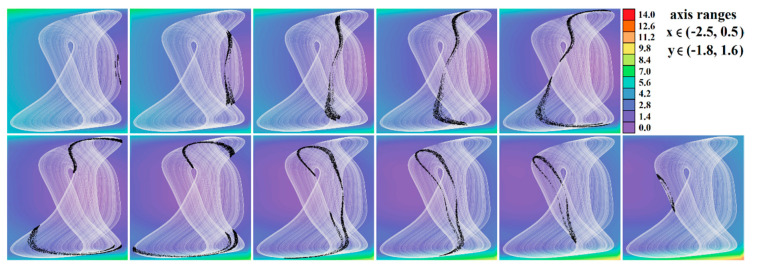
Horizontal state space slices given by plane *z = const*. providing rainbow scaled dynamical energy distribution of the typical chaotic attractors of case **Ψ**_7_ system (white trajectory), and associated Poincaré sections (black dots). Individual figures sorted from left to right and up to down are given by: *z =* −1.4, *z =* −1, *z =* −0.5, *z =* −0.2, *z =* 0, *z =* 0.2, *z =* 0.5, *z =* 1, *z =* 1.5, *z =* 1.8, *z =* 2.2, and *z =* 2.7.

Figure 4, Figure 5, Figure 6, Figure 7, Figure 8, Figure 9 and Figure 10 demonstrate the distribution of dynamic energy through the state space for individual cases **Ψ**_1–7_ (see Table 1). Here, red color denotes high kinetic energy, green marks average local energy and magenta indicates a very low local energy. Numerical values associated with these rainbow scaled plots normalized to unity time intervals are also provided. Initial conditions and time step were kept the same as for the analysis given in Figure 2; both with integration input parameters and a set of the initial conditions. Firstly, note that strange attractors occupy different sized volumes in the state space. Therefore, each plotted high-resolution plane has different axis ranges but uniform step size 0.01; concrete boundaries can be found within descriptors of the individual figures. For system cases **Ψ**_1_ and **Ψ**_4_, it is obvious that average dynamic energy rises with the absolute value of state variable *z*. Using visualized plots, geometrical similarity between system cases **Ψ**_1_ and **Ψ**_4_ can be observed. Finally, strange attractors primarily do not evolve within areas with very high or low normalized energy. Along with kinetic energy distributions, Poincaré return maps for the horizontal slices of the state space (*z* = const) are visualized. Obviously, geometrical shapes of generated strange attractors are distinct and attractors are dense in the state space, outside regions of a high local differential growth. If indicated in the plot, vector field symmetry causes the strange attractor to be mirrored with respect to the zero plane (*z* = 0) and Poincaré sections could only be provided for upper or lower half state space (system cases **Ψ**_2,3,6_).

Table 3 provides calculated values that can quantify the complexity of typical strange attractors generated by the subclasses of a class C amplifier cell with a single transistor **Ψ**_1_ up to **Ψ**_7_. The first flow quantifier is the largest Lyapunov exponent (LLE) calculated using the mathematical model, see [30,31] for an overall description and algorithm explanation. Based on the spectrum of one-dimensional Lyapunov exponents (real numbers calculated with transient behavior omitted), the so-called Kaplan–Yorke dimension (KYD) of a generated strange attractor is established [32,33]. Capacity dimension (CD) of the state space attractor established by using the box counting method [34] is also provided. Mentioned flow quantifiers adapted for third-order dynamical systems can be calculated as follows:

(17) $$\mathrm{LLE}=\underset{t\to \infty }{\lim}\underset{\left|\delta Z_{0}\right|\to 0}{\lim}\frac{\left|\delta Z\left(t\right)\right|}{\left|\delta Z_{0}\right|},\;\mathrm{KYD}=2+\frac{LE_{1}+LE_{2}}{LE_{3}}\;,\;\mathrm{CD}=\underset{\varepsilon \to 0}{\lim}\frac{\ln N\left(\varepsilon \right)}{\ln1/\varepsilon }\;,$$

where LLE is calculated using flow linearization and change in cube volume after small integration step δ**Z**(*t*), *LE*_1_ > *LE*_2_ > *LE*_3_ are one-dimensional Lyapunov exponents arranged in a decreasing order, and *N*(*ε*) is the number of cubes with edge *ε* required to fully cover the inspected state attractor.

The self-similarity of patterns of time sequences with different lengths produced by the inspected chaotic systems is measured using the so-called approximate entropy (ApEn). The approach for how to deal with this problem including its algorithmizing in a Matlab environment is provided in several research papers, for example, [35,36]. The ApEn routine has several input parameters, and the number provided in Table 3 is the biggest calculated value of ApEn for the particular case **Ψ**. Here, a data sequence with a length of 1000 samples, embedding dimension 3 and time delay 1, was adopted. Table 3 can be roughly evaluated as follows: second case **Ψ**_2_ can be considered as the most unpredictable system with the most complex geometric structure of the strange attractor, while cases **Ψ**_1_, **Ψ**_5_ and **Ψ**_7_ produce chaotic waveforms with the most significant entropic properties. These are probably a good choice for applications in secure communications, chaos-based modulation/masking techniques, etc.

**Figure 11:**
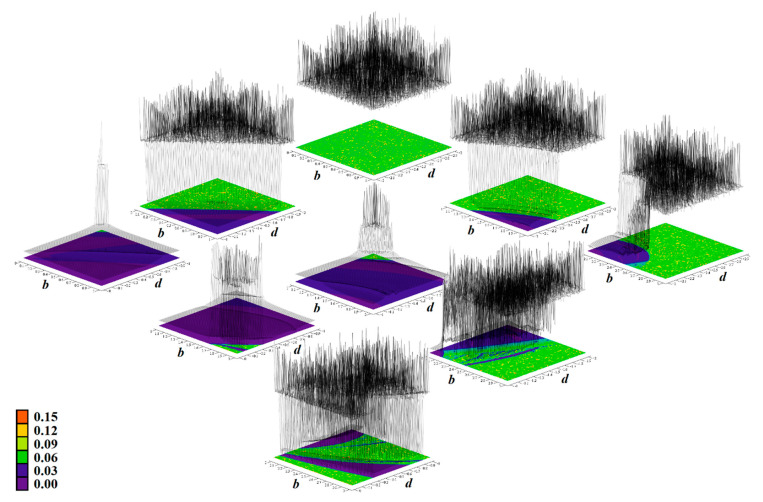
Rainbow scaled surface-contour plot of the largest Lyapunov exponent as two-dimensional function of nonlinear feedback, calculated for **Ψ**_1_ case of chaotic circuit and total range of parameters is *a*∈(0, 3) and *b*∈(−3, 0).

**Figure 12:**
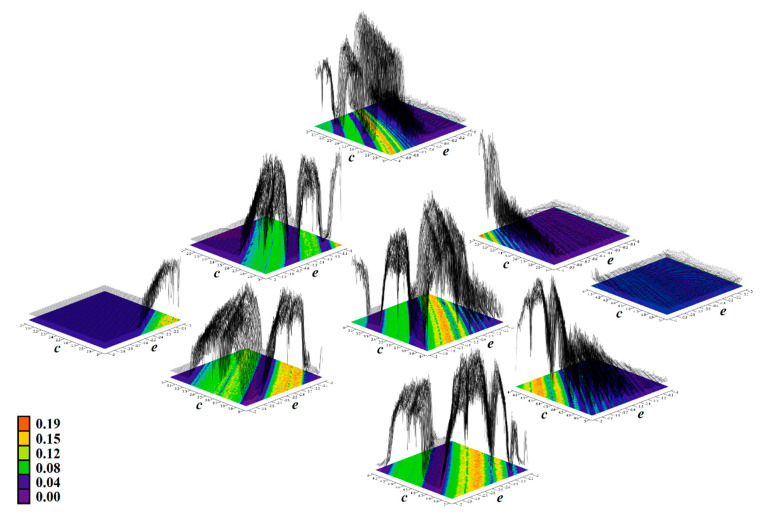
Rainbow scaled surface-contour plot of the largest Lyapunov exponent as two-dimensional function of nonlinear feedback, area covering both **Ψ**_2_, **Ψ**_5_, and **Ψ**_7_ with dissipation coefficient *y*_11_ = 0.4, total range of parameters is *c*∈(2, 5) and *e*∈(−3, 0).

**Figure 13:**
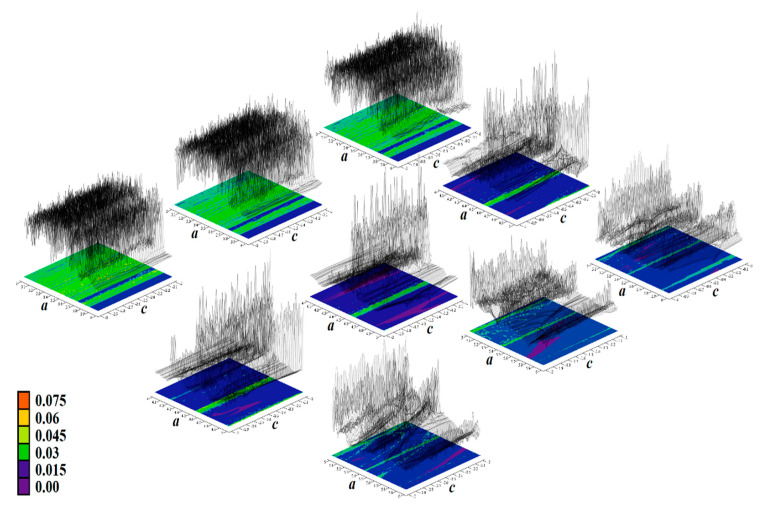
Rainbow scaled surface-contour plot of the largest Lyapunov exponent as two-dimensional function of nonlinear feedback, area covering case **Ψ**_3_ and total range of parameters is *a*∈(3, 6) along with *b*∈(−3, 0).

**Figure 14:**
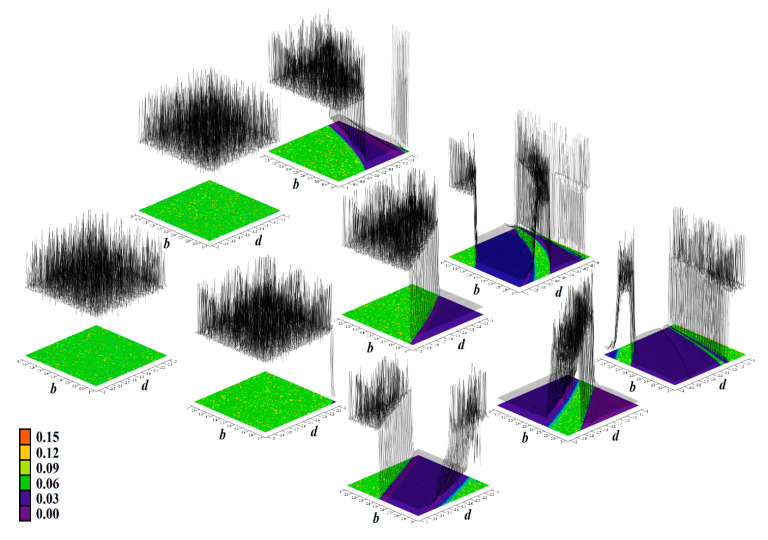
Rainbow scaled surface-contour plot of the largest Lyapunov exponent as two-dimensional function of nonlinear feedback, area covering case **Ψ**_4_ and total range of parameters is *b*∈(0, 3) along with *d*∈(−3, 0).

**Figure 15:**
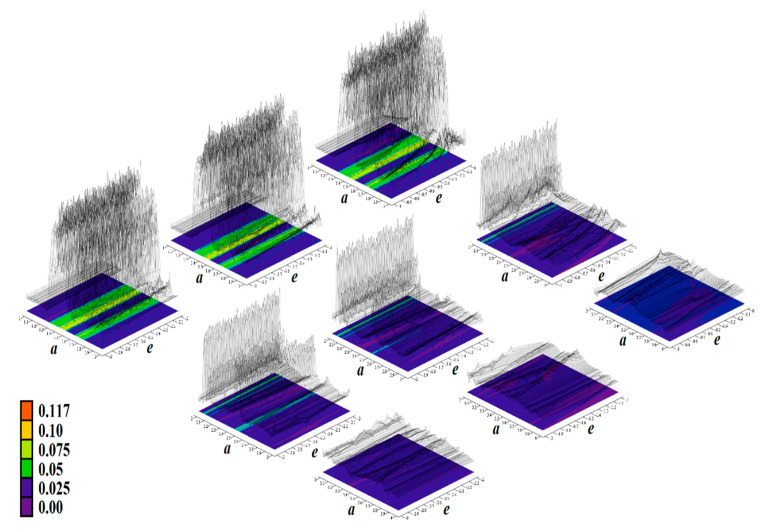
Rainbow scaled surface-contour plot of the largest Lyapunov exponent as two-dimensional function of nonlinear feedback, area covering case **Ψ**_6_ and total range of parameters is *a*∈(1, 4) along with *e*∈(−3, 0).

Figure 11, Figure 12, Figure 13, Figure 14 and Figure 15 prove that the regions of chaos for individual cases **Ψ** are wide enough such that the geometry of desired strange attractors will be structurally stable and experimentally observable—consult paper [37] for details. This is important since real values of the circuit components fluctuate with time, ambient temperature and heating, and they are inaccurate due to the fabrication tolerances, etc. Moreover, these effects neither compensate each other nor have mutual correlations. Color scale (legends with values of LLE are provided directly within individual plots) corresponds to the solution of a dynamical system (1) as follows: red denotes unbounded solution, yellow and green represent strong and weak chaos, respectively, blue color marks areas where the ω-limit set is periodic solution, and magenta highlights areas where trajectory is slowly attracted to the fixed point. In this case, final time was extended to 5000 s and the data sequence for calculation was stored after 500 s to remove short as well as long transients. To obtain sufficient accuracy (high resolution) of all plots, the parameter step was decreased to 0.01 such that each plot contained 101 × 101 = 10,201 points. Vertical axis is provided using a linear scale starting with zero. Note that system case **Ψ**_3_ has a very narrow parameter subspace that leads to the geometrically stable predefined chaotic attractor. On the other hand, nominal values of internal parameters of system case **Ψ**_1_ can be adjusted such that the prescribed strange attractor is very robust and cannot be violated by various imperfections during circuit construction.

**Figure 16:**
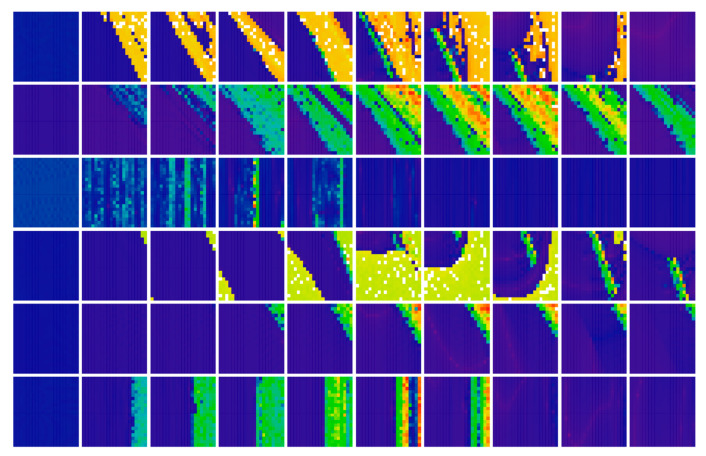
Rainbow scaled plot showing flow quantification for the individual cases **Ψ**_1–6_ (rows 1 to 6) of chaotic class C amplifier and increased value of system dissipation *y*_11_ = 0, 0.1, 0.2, 0.3, 0.4, 0.5, 0.6, 0.7, 0.8, 0.9 (columns from left to right), see text for better clarification.

Figure 16 graphically demonstrates that randomness of generated chaotic motion disappears for both decreased and increased dissipation of the analyzed mathematical model, represented by the input admittance of bipolar transistor *y*_11_. However, corresponding patterns are different for the particular cases **Ψ**_1–6_. To demonstrate this property, dynamical flow was quantified and divided into the following classes: unbounded solution (white), strong chaos (red), weak chaos (yellow), limit cycle (green) and fixed point solution (blue). The parameter step for all plots is chosen uniformly as 0.05, axis scale for case **Ψ**_1_ is *b*∈(2, 3), *d*∈(−2, −1), for second system **Ψ**_2_ it is *c*∈(2, 3), *e*∈(−2, −1), third case **Ψ**_3_ has axis ranges *a*∈(4, 5), *c*∈(−2, −1), fourth case **Ψ**_4_ is characterized in ranges *b*∈(2, 3), *d*∈(−3, −2), results for fifth case **Ψ**_5_ are given in ranges *c*∈(2, 3), *e*∈(−3, −2), and for the case **Ψ**_6_ it is *a*∈(1, 2), *e*∈(−1.5, −0.5). For this kind of analysis, low resolution plots with 21 × 21 = 441 points have been calculated.

**Figure 17:**
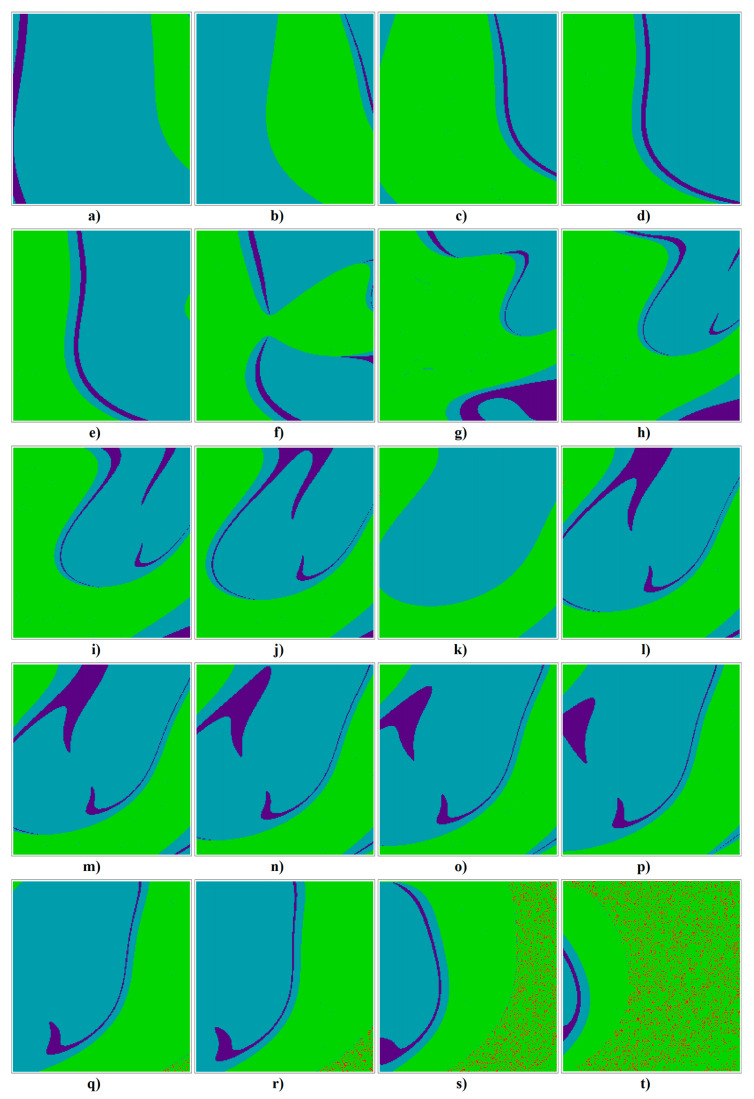
Colored basins of attraction, individual slices are the horizontal planes: (**a**) z0 = −3, (**b**) z0 = −2.5, (**c**) z0 = −2, (**d**) z0 = −1.5, (**e**) z0 = −1.25, (**f**) z0 = −1, (**g**) z0 = −0.75, (**h**) z0 = −0.5, (**i**) z0 = −0.3, (**j**) z0 = −0.1, (**k**) z0 = 0.0, (**l**) z0 = 0.1, (**m**) z0 = 0.2, (**n**) z0 = 0.3, (**o**) z0 = 0.4, (**p**) z0 = 0.5, (**q**) z0 = 0.75, (**r**) z0 = 1.0, (**s**) z0 = 1.5, and (**t**) z0 = 2.

Figure 17 illustrates geometric structures in the state space associated with individual attractors for case **Ψ**_1_ transistor cell. Plots are calculated for ranges *x*_0_∈(−1, 1), *y*_0_∈(−1, 1) and each plot contains 201 × 201 = 40,401 sets of initial conditions. Note that neighborhood of equilibrium is not a part of the basin of attraction for the chaotic attractor. Basins of attraction are colored as follows: unbounded solution (red), strange attractor (green), limit cycle (light blue) and fixed point solution (dark blue).

## 4. Design of Flow-Equivalent Chaotic Oscillator

Verification through practical experiment belongs to the common standard for the presentation of a new chaotic dynamical system. It is widely adopted that the observability of strange attractors represents satisfactory proof of the robustness of desired dynamics.

Lumped circuit synthesis based on a prescribed mathematical model is a problem that can be easily solved using several different approaches. One of the most popular methods is based on an integrator block schematic, where basic mathematical operations are performed by three types of the two-port building blocks: inverting summing integrators, differential amplifiers and blocks having piecewise-linear or polynomial transfer curve. Each mentioned operation requires at least a single active element, usually a voltage feedback operational amplifier. The main drawback of this concept is evident: the necessity of using many active elements and a rather high power consumption. An integrator based kind of circuit realization is possible in three operational regimes: the most preferred is voltage-mode concept [38], current-mode is usually dedicated for the higher frequency bands [39] and mixed-mode circuits.

**Figure 18:**
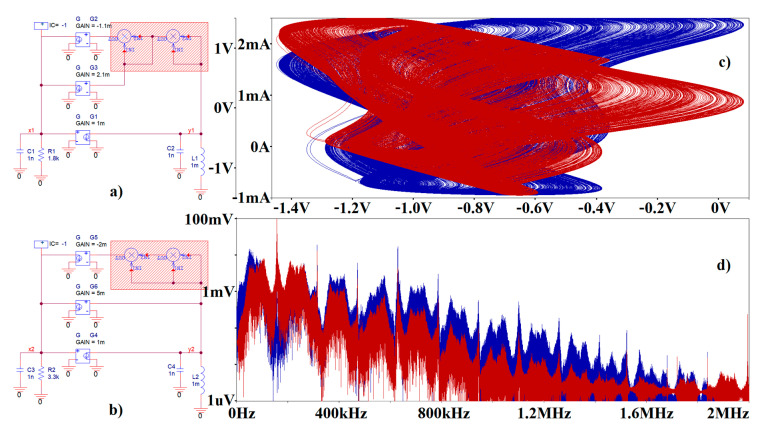
Idealized circuit realization of a chaotic system with the emulated bipolar transistor stage: (**a**) case **Ψ**_1_ system with parameters taken from Table 1, (**b**) case **Ψ**_3_ system with parameter set taken from Table 1, (**c**) Monge projections *v_y_*_1_ vs. *v_x_*_1_ (blue) and *i_L_*_1_ vs. *v_x_*_1_ (red), (**d**) frequency spectrum of generated signal *v_x_*_1_ (blue) and *v_y_*_1_ (red). Red areas represent polynomial feedback transfer functions.

It is worth nothing that the Orcad Pspice circuit simulator was used for the pre-validation of designed chaotic oscillators. Figure 18 shows maximally idealized case **Ψ**_1_ and **Ψ**_3_ systems with impedance norm 10^3^ and frequency norm 10^6^. Of course, having ideal voltage-controlled current-sources (G) and ideal voltage multiplication blocks (MULT), both norms can be arbitrary; only simulation profile setup needs to be adjusted accordingly. In our case, final time was set to 10 ms (to visualize the robust strange attractor), maximum allowed time step was reduced to 1 μs (to demonstrate the density of the strange attractor) and pseudo-components IC = −1V (IC1) served to set nonzero initial conditions into the circuit. In real circuitry, the injection of certain initial conditions is a much more complicated task.

**Figure 19:**
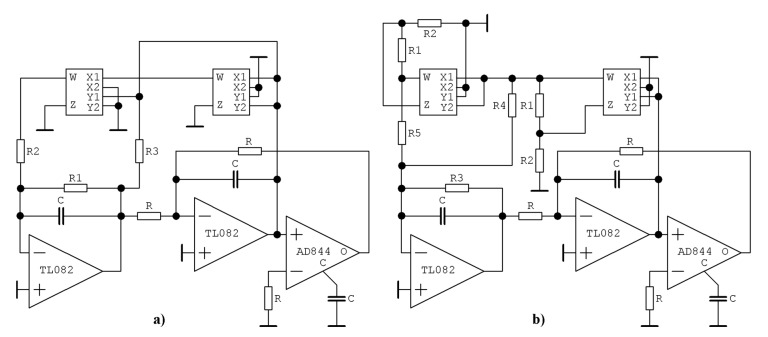
Chaotic system with emulated bipolar transistor stage: (**a**) circuit realization of differential Equations (1) and (3) and parameter set **Ψ**_3_ taken from Table 1, (**b**) circuit implementation of Equations (1) and (3) and parameter set **Ψ**_1_ or **Ψ**_4_ taken from Table 1.

**Figure 20:**
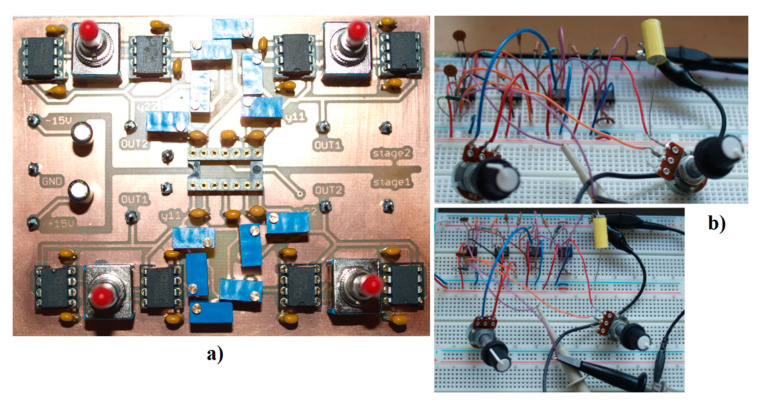
Photos captured during experimental investigation: (**a**) PCB showing two two-ports where transconductances *y*_12_ and *y*_21_ are polynomials up to the fourth-order, (**b**) two views onto breadboard with designed chaotic oscillator based on generalized class C amplifier.

Voltage-mode realizations ready for simulation/construction are provided in Figure 19 and these circuits undergo deep experimental verification. Supply voltage is symmetrical ±15 V. The designed oscillator consumes two cheap voltage feedback operational amplifiers TL082 (in a single package), one current-feedback operational amplifier with compensation node (denoted by letter C) AD844 and two four-quadrant analog multipliers AD633. Schematic and associated realization using a breadboard are provided by means of Figure 20. The first designed chaotic oscillator (Figure 19a) is described by following ordinary differential equations:

(18) $$\frac{d}{dt}v_{1}=-\frac{v_{1}}{R_{1}C}-\frac{v_{2}}{R_{3}\cdot C}+K^{2}\frac{v_{2}^{3}}{R_{2}C}\;,\;\frac{d}{dt}v_{2}=-\frac{v_{1}}{R\cdot C}-\frac{v_{3}}{R\cdot C}\;,\;\frac{d}{dt}v_{3}=\frac{v_{2}}{R\cdot C}\;,\;$$

where state vector transforms into **x** = (*v*_1_, *v*_2_, *v*_3_)^T^ and *K* = 0.1 is the internally trimmed transfer constant of AD633. Note that this chaotic system models the behavior of function (3) with nonzero values *a* and *c*, whereby other terms are zero.

Similarly, the second dynamical system is able to model differential equations with the polynomial function (3) with nonzero values of coefficients *b* and *d*. The set of differential equations is:

(19) $$\frac{d}{dt}v_{1}=-\frac{v_{1}}{R_{3}C}-\left(K+\frac{R_{2}}{R_{1}+R_{2}}\right)\frac{v_{2}^{2}}{R_{4}C}+{\left(K+\frac{R_{2}}{R_{1}+R_{2}}\right)}^{3}\frac{v_{2}^{4}}{R_{5}C}\;,\;\frac{d}{dt}v_{2}=-\frac{v_{1}}{R\cdot C}-\frac{v_{3}}{R\cdot C}\;,\;\frac{d}{dt}v_{3}=\frac{v_{2}}{R\cdot C}\;,\;$$

where argument in brackets can be chosen advantageously such that equality *K* + *R*_2_/(*R*_1_ + *R*_2_) = 1 holds. The fundamental time constant of this circuit is *τ* = *R*⋅*C* = 10^4^⋅10^−8^ = 100 μs, but the main frequency components can be shifted toward the GHz band easily by appropriate frequency rescaling. Considering the normalized numerical values provided in Table 1, impedance rescaling 10^4^ and frequency norm 10^8^ circuit components for (18) with parameter set **Ψ**_3_ are: *C* = 10 nF, *R* = 10 kΩ, *R*_1_ = 33 kΩ, *R*_2_ = 50 Ω, and *R*_3_=2 kΩ. Analogically, circuit components for (19) with parameter set **Ψ**_2_ (**Ψ**_4_) are the following: *C* = 10 nF, *R* = 10 kΩ, *R*_1_ = 1 kΩ, *R*_2_ = 9 kΩ, *R*_3_ = 18 kΩ (25 kΩ), *R*_4_ = 4.8 kΩ (3.7 kΩ), and *R*_5_ = 91 Ω (50 Ω). Figure 20a shows the PCB (Printed Circuit Board) of two uncoupled two-ports modeled by the adjustable admittance parameters. While input and output admittance is linear and represented by a variable resistor, trans-admittance *y*_12_ and *y*_21_ are polynomials up to the fourth order. The unoccupied socket is dedicated for integrated circuit TL084 (four operational amplifiers in a single package), or its empty pins can be used to connect PCB with the breadboard. PCB is designed such that a user can use switches to change signs of all coefficients of the polynomial trans-conductance *y*_12_(*v*_2_) and/or *y*_21_(*v*_1_). Figure 20b demonstrates the simplicity of the designed chaotic system.

## 5. Experimental Verification

**Figure 21:**
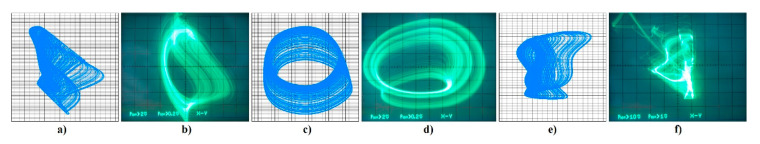
Dynamical system (1) with (3) and values **Ψ**_3_ from Table 1, Comparison between numerical integration process (blue) and laboratory experiment (green): (**a**,**b**) *v*_1_ vs. *v*_3_ plane, (**c**,**d**) *v*_2_ vs. *v*_3_ plane, (**e**,**f**) *v*_1_ vs. *v*_2_ plane.

**Figure 22:**
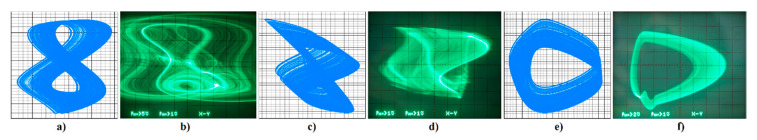
Dynamical system (1) with (3) and values **Ψ**_1_ from Table 1, comparison between numerical integration process (blue) and laboratory experiment (green): (**a**,**b**) *v*_1_ vs. *v*_3_ plane, (**c**,**d**) *v*_1_ vs. *v*_2_ plane, (**e**,**f**) *v*_2_ vs. *v*_3_ plane.

**Figure 23:**
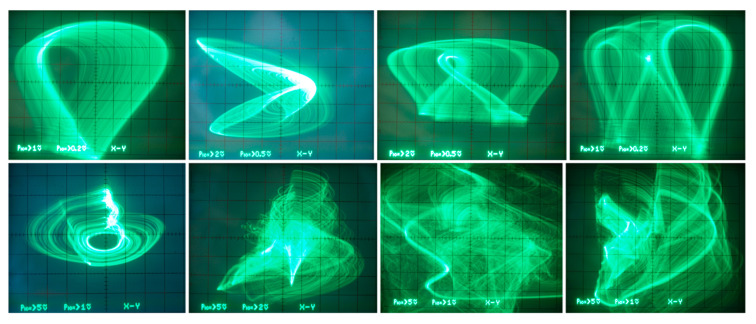
Different Monge projections of strange attractors not mutually connected with numerical analysis of generalized chaotic class C amplifier.

Selected strange attractors observed during experimental verification are provided in Figure 21, Figure 22 and Figure 23. In the first two cases, individual plane projections captured by an oscilloscope are compared with numerically integrated results and Equations (1) and (3) for parameter set **Ψ**_3_ and **Ψ**_1_, respectively—values are given in Table 1. A uniform 100 mV grid is used for numerical integration results. For the latter case, numerical mirrors of the visualized strange attractors are not provided. During measurement, strong sensitivity of type of the steady state to the initial conditions imposed into the chaotic oscillator has been confirmed. Nevertheless, goodish correspondence between theory and practical experiment was achieved. The route-to-chaos scenario can be traced via the shaping of nonlinear *y*_12_(*v*_2_) function, namely by variable resistors *R*_4_ and *R*_5_ in Figure 19b. To obtain classes of the chaotic system characterized by sets **Ψ**_2_, **Ψ**_5_, **Ψ**_6_, and **Ψ**_7_, additional AD633 is necessary. However, a major part of the proposed oscillator remains unchanged.

**Figure 24:**
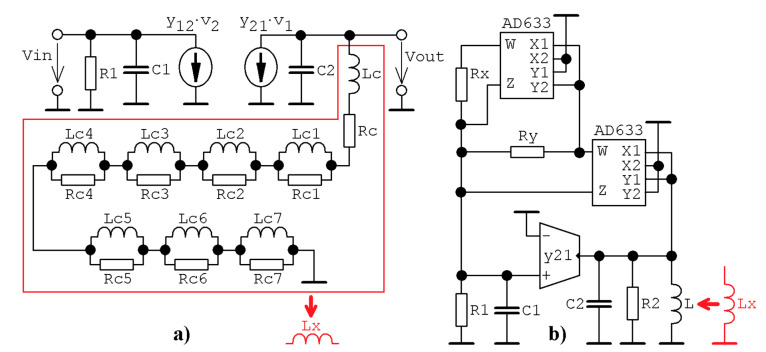
Two alternative lumped circuitry implementations of class C potentially chaotic amplifier: (**a**) principal schematic of dynamical system with passive approximated fractional-order inductor, (**b**) realization based directly on the state model (20).

Different realization of the chaotic oscillator offers the principal schematic given in Figure 24b. This system is described by following a set of ordinary differential equations:

(20) $$C_{1}\frac{d}{dt}v_{1}=-\frac{v_{1}}{R_{1}}-\frac{K}{R_{y}}v_{2}^{2}+\frac{K^{2}}{R_{x}}v_{2}^{4}\;,C_{2}\frac{d}{dt}v_{2}=-i_{L}-y_{21}\cdot v_{1}\;,L\frac{d}{dt}i_{L}=v_{2}\;,$$

where the forward transconductance *y*_21_ is realized by a single-input single-output operational trans-conductance amplifier. Nonlinear transfer function is implemented by couple (third-order polynomial for **Ψ**_3_, fourth-order polynomial for **Ψ**_1_ and **Ψ**_4_) or three (fifth-order polynomial to reach sets of parameters **Ψ**_2_, **Ψ**_5_ and **Ψ**_6_) AD633.

Speaking in terms of commercially available active devices, trans-conductance amplifiers are available as LM13700, LT1228, MAX435, or diamond transistors OPA660, etc. Note that a nonlinear two-port needs to work in trans-admittance regime, i.e., with input voltage and output current. If both impedance and frequency scaling factors could be arbitrary real numbers, we experience two degrees of freedom for the calculation of inductance and capacitance. Therefore, the parallel inductor-capacitor resonant tank could be arbitrary as well, i.e., associated with an audio amplifier, an active part of a sensor element, a model of piezo-element, a matching subcircuit, etc.

### Fractional-Order Chaotified Class C Amplifier

Now assume that the transistor is loaded by the non-integer order LC tank. Fractionality will be represented by the presence of a fractional-order (FO) inductor. This FO inductor will be approximated in the frequency domain by a more complicated network [40] with a phase frequency response of impedance rippled around ideal value 90°*γ*. Mentioned approximation should be valid at least in a finite frequency range that corresponds to the desired chaotic signal generated by the FO chaotic class C amplifier. In our case, frequency range turns to be from 1 Hz up to 2 MHz (see also Figure 18) and the minimal complexity of the FO inductor is 7 (number of required resistor-inductor sections). Note that the impedance of the RL approximation circuit tends to *R_c_* for very low frequencies and reaches to infinity for high frequencies. A set of ordinary differential equations that describes circuitry given in Figure 24b with an FO inductor *L_x_* with seven sections is as follows:

(21) $$\frac{d}{dt}v_{1}=-\frac{v_{1}}{R_{1}\cdot C_{1}}-\frac{K}{R_{y}\cdot C_{1}}\;v_{2}^{2}+\frac{K^{2}}{R_{x}\cdot C_{1}}\;v_{2}^{4},\;\frac{d}{dt}v_{2}=-\frac{v_{2}}{R_{2}\cdot C_{2}}-\frac{y_{21}}{C_{2}}\cdot v_{1}-\frac{1}{C_{2}}\cdot i_{L_{c}}\;,\;\frac{d}{dt}i_{L_{c}}=\frac{v_{2}}{L_{c}}-\frac{R_{c}}{L_{c}}\cdot i_{Lc}-\frac{1}{L_{c}}\cdot \sum_{k=1}^{7}R_{ck}\left(i_{L_{c}}-i_{L_{ck}}\right)\;,\;\frac{d}{dt}i_{L_{ck}}=\frac{R_{ck}}{L_{ck}}\left(i_{L_{c}}-i_{L_{ck}}\right)\;,$$

where *k* = 1, …, 7.

Speaking in terms of FO network analysis, this is the place where conventional circuit-oriented simulation software such as Orcad Pspice can be utilized. A gallery of passive ladder FO capacitors calculated for important decimal orders between zero and one can be found in paper [41]. Structures proposed in this paper have been optimized from the viewpoint of low phase error (less than 1.5°) and wide frequency range (from 3 Hz up to 3 MHz). By following the duality principle, these findings can be extended and FO inductors for orders 9/10 (Table 4), 8/9 (Table 5), 4/5 (Table 6), and 3/4 (Table 7) are presented. Numerical values provided in these tables lead to FO inductors having unity pseudo-inductance, i.e., the module of impedance measured at the specific frequency *f*_0_ = 1/(2*π*) Hz is 1 *s*^1−α^/F, where *α* represents math order.

Author encouragement for interested readers: please do not hesitate to contact me (via email) if a specific mathematical order, frequency range, approximation network complexity or different phase accuracy of the FO capacitor and/or inductor is required.

## 6. Discussion

This paper brings an example of an electronic circuit for which, under very specific circumstances, the circuit can switch from regular into chaotic behavior. Conditions leading to chaotic motion can be summarized as follows:

1. General mathematical models analyzed in this paper (3) and (10) contain normalized values of all accumulation elements. After optimization, to observe strange attractors, resulting parasitic capacitance as well as capacitance and inductance located within the LC resonant tank are of comparable orders. Therefore, parasitic accumulation elements turn into functional. This fact increases the intrinsic number of degrees of freedom and forces a naturally non-chaotic analogue building block to behave chaotically. Because of the internal structure of bipolar transistors commonly used in class C amplifiers, this kind of motion is possible only for assumed high-frequency operation. In practice, generated chaotic waveform can be easily misinterpreted as noise.
2. The second condition for chaos evolution is the presence of a specific local nonlinear feedback. In the mathematical model of the analyzed dynamical system, either polynomial or PWL scalar function is the only nonlinearity.
3. The third specific property of a bipolar transistor is linear backward trans-conductance. Its value is non-zero and relatively large.

## 7. Conclusions

This manuscript admits the parasitic properties of a bipolar transistor to be the working accumulation elements; base-emitter capacitance is mandatory, while collector-emitter capacitance may not be present. There is one consequence resulting from this research: chaos belongs to the natural behavior of sub circuits that contain at least one bipolar transistor, although the expected working regime may seem rather hypothetical for a practically oriented design engineer. This statement agrees with the conclusion reached in paper [42], where the JFET element is the single locally active element while the coil is the passive one. In our case, fingerprints of a bipolar transistor can be found in both folding and stretching mechanisms of a vector field. Additionally, a set of ordinary differential equations together with the six different sets of internal parameters proposed in this paper can be considered as a new chaotic dynamical system. This system is a member of autonomous deterministic systems with a single center-type stable equilibrium point. Strange attractors observed in the frame of numerical investigations are in good accordance with those captured as oscilloscope screenshots using a flow-equivalent circuit.

This paper leaves significant space for further research, for example, to find system parameters close to the common operation of a single transistor stage where:

1. Parasitic capacitors are working ones,
2. Nonlinearity is typical for a large signal model of a bipolar transistor,
3. An additional degree of freedom is presented because driving force (processed signal) changes the operational point of an analyzed circuit.

The results presented in this work are strictly associated with a single-stage class C amplifier with a single bipolar transistor. It is well known that the probability of chaos rises with the total order of a circuit. Therefore, the existence of various strange attractors can be expected for electronic systems with several transistors, such as Darlington circuits, cascode connections, current mirrors, multi-stage amplifiers, etc. Much more complex behavior including higher-dimensional chaos and hyper-chaos can be expected if several transistors coexist and interact.

## Figures and Tables

**Table 1:** Numerical values of internal parameters of system (3) with mathematical orders α = β = γ = 1 that result in robust chaotic motion.

Case	*y*_11_	*a*	*b*	*c*	*d*	*e*
**Ψ**_1_	0.56	0	2.1	0	−1.1	0
**Ψ**_2_	0.50	0	0	3	0	−1.5
**Ψ**_3_	0.30	5	0	−2	0	0
**Ψ**_4_	0.40	0	2.7	0	−2	0
**Ψ**_5_	0.30	0	0	3	0	−2
**Ψ**_6_	0.50	2	0	0	0	−0.5
**Ψ**_7_	0.40	0	0	2	0	–1

**Table 2:** Numerical values of internal parameters of system (10) with mathematical orders α = β = γ = 1 and either (11) or (12) that result in structurally stable chaotic motion (NA means Not Available).

Case	*y*_11_	*ϕ*	*ϕ*_1_	*ϕ*_2_	*ρ*_0_	*ρ*_1_	*ρ*_2_
**Ψ**_8_	0.56	1.1	NA	NA	1	−4.3	NA
**Ψ**_9_	0.5	NA	0.3	1.1	0.3	2	−7
**Ψ**_10_	0.3	NA	0.6	1.18	4.6	0.6	−9.9
**Ψ**_11_	0.3	NA	0.4	1	0.2	1.5	−9.5

**Table 3:** Geometric and time-domain features of generated typical strange attractors.

Case	LLE	KYD	CD	ApEn
**Ψ**_1_	0.071	2.113	2.15	0.539
**Ψ**_2_	0.156	2.239	2.24	0.558
**Ψ**_3_	0.045	2.132	2.14	0.440
**Ψ**_4_	0.020	2.050	2.10	0.503
**Ψ**_5_	0.069	2.186	2.20	0.564
**Ψ**_6_	0.047	2.081	2.15	0.518
**Ψ**_7_	0.050	2.160	2.13	0.620

**Table 4:** Numerical values of fully passive series-parallel circuit realization of fractional-order (FO) inductor with mathematical order 9/10, i.e., phase shift between voltage and current 81°.

*R_a_*	*R*_1_	*R*_2_	*R*_3_	*R*_4_	*R*_5_	*R*_6_	*R*_7_
0.6 Ω	3.3 Ω	22.7 Ω	153 Ω	1031 Ω	6944 Ω	46.7 kΩ	313 kΩ
*L_a_*	*L*_1_	*L*_2_	*L*_3_	*L*_4_	*L*_5_	*L*_6_	*L*_7_
144 mH	120 mH	98 mH	79 mH	64 mH	52 mH	42 mH	37 mH

**Table 5:** Numerical values of fully passive series-parallel circuit realization of FO inductor with mathematical order 8/9, i.e., phase shift between voltage and current 80°.

*R_a_*	*R*_1_	*R*_2_	*R*_3_	*R*_4_	*R*_5_	*R*_6_	*R*_7_
1 Ω	6.3 Ω	44.4 Ω	319 Ω	2286 Ω	16.4 kΩ	118 kΩ	833 kΩ
*L_a_*	*L*_1_	*L*_2_	*L*_3_	*L*_4_	*L*_5_	*L*_6_	*L*_7_
203 mH	230 mH	194 mH	152 mH	120 mH	93 mH	73 mH	62 mH

**Table 6:** Numerical values of fully passive series-parallel circuit realization of FO inductor with mathematical order 4/5, i.e., phase shift between voltage and current 72°.

*R_a_*	*R*_1_	*R*_2_	*R*_3_	*R*_4_	*R*_5_	*R*_6_	*R*_7_
1.1 Ω	4.7 Ω	25.8 Ω	141 Ω	769 Ω	4184 Ω	22.7 kΩ	133 kΩ
*L_a_*	*L*_1_	*L*_2_	*L*_3_	*L*_4_	*L*_5_	*L*_6_	*L*_7_
35 mH	237 mH	155 mH	101 mH	66 mH	43 mH	28 mH	22 mH

**Table 7:** Numerical values of fully passive series-parallel circuit realization of FO inductor with mathematical order 3/4, i.e., phase shift between voltage and current 67.5°.

*R_a_*	*R*_1_	*R*_2_	*R*_3_	*R*_4_	*R*_5_	*R*_6_	*R*_7_
1.2 Ω	4.5 Ω	22 Ω	108 Ω	526 Ω	2591 Ω	12.7 kΩ	55.6 kΩ
*L_a_*	*L*_1_	*L*_2_	*L*_3_	*L*_4_	*L*_5_	*L*_6_	*L*_7_
13 mH	210 mH	132 mH	78 mH	46 mH	27 mH	16 mH	10 mH
